# The Marine Fungal Metabolite, AD0157, Inhibits Angiogenesis by Targeting the Akt Signaling Pathway

**DOI:** 10.3390/md12010279

**Published:** 2014-01-16

**Authors:** Melissa García-Caballero, Librada Cañedo, Antonio Fernández-Medarde, Miguel Ángel Medina, Ana R. Quesada

**Affiliations:** 1 Department of Molecular Biology and Biochemistry, Faculty of Sciences, University of Málaga, Campus de Teatinos, Málaga 29071, Spain; E-Mails: melissa@uma.es (M.G.-C.); medina@uma.es (M.A.M.); 2 Unidad 741 de CIBER “de Enfermedades Raras”, University of Málaga, Málaga E-29071, Spain; 3 International Campus of Excellence Andalucía Tech, Málaga 29071, Spain; 4 Biomar Microbial Technologies, Parque Tecnológico de León, Parcela M-10.4, Armunia (León) 24009, Spain; E-Mails: acanedohe@terra.es (L.C.); a.fernandez@biomar.co (A.F.-M.)

**Keywords:** AD0157, angiogenesis, cancer, apoptosis, marine drug, pyrrolidinedione

## Abstract

In the course of a screening program for the inhibitors of angiogenesis from marine sources, AD0157, a pyrrolidinedione fungal metabolite, was selected for its angiosupressive properties. AD0157 inhibited the growth of endothelial and tumor cells in culture in the micromolar range. Our results show that subtoxic doses of this compound inhibit certain functions of endothelial cells, namely, differentiation, migration and proteolytic capability. Inhibition of the mentioned essential steps of *in vitro* angiogenesis is in agreement with the observed antiangiogenic activity, substantiated by using two in vivo angiogenesis models, the chorioallantoic membrane and the zebrafish embryo neovascularization assays, and by the *ex vivo* mouse aortic ring assay. Our data indicate that AD0157 induces apoptosis in endothelial cells through chromatin condensation, DNA fragmentation, increases in the subG1 peak and caspase activation. The data shown here altogether indicate for the first time that AD0157 displays antiangiogenic effects, both *in vitro* and *in vivo*, that are exerted partly by targeting the Akt signaling pathway in activated endothelial cells. The fact that these effects are carried out at lower concentrations than those required for other inhibitors of angiogenesis makes AD0157 a new promising drug candidate for further evaluation in the treatment of cancer and other angiogenesis-related pathologies.

## 1. Introduction

Angiogenesis, a physiological process involving the generation of new capillaries from pre-existing vessels, is strictly controlled by a balance of stimulators and inhibitors, being restricted in adults to some processes related to the reproductive cycle and wound repair. However, angiogenesis is now widely recognized as one of the hallmarks of cancer, a crucial step in the transition of tumors from a dormant state to a malignant state, and playing an essential role in tumor growth, invasion and metastasis [1]. Furthermore, a continuously increasing number of other non-neoplastic diseases are being related to an upregulated angiogenesis. They include diabetic retinopathy, age-related macular degeneration, hemangioma, arthritis and psoriasis, among others [2]. Currently, angiogenesis inhibitors are likely to change the face of medicine, arising as an attractive approach for the treatment of cancer and other angiogenesis-dependent diseases, with some antiangiogenic compounds approved by the Food and Drug Administration (FDA) for the treatment of cancer, blindness and other angiogenesis-dependent diseases, encouraging expectations in their therapeutic potential [3,4].

In response to the angiogenic stimulus, the normally quiescent endothelial cells become activated and undergo a series of phenotypic changes, including the release of proteases that will allow them to degrade the extracellular matrix and migrate. Activated endothelial cells will proliferate and avoid apoptosis, which could be triggered by the loss of survival signals and, finally, will differentiate, rendering a new capillary. Any of these steps could be a target for the pharmacological inhibition of angiogenesis [5].

**Figure 1:**
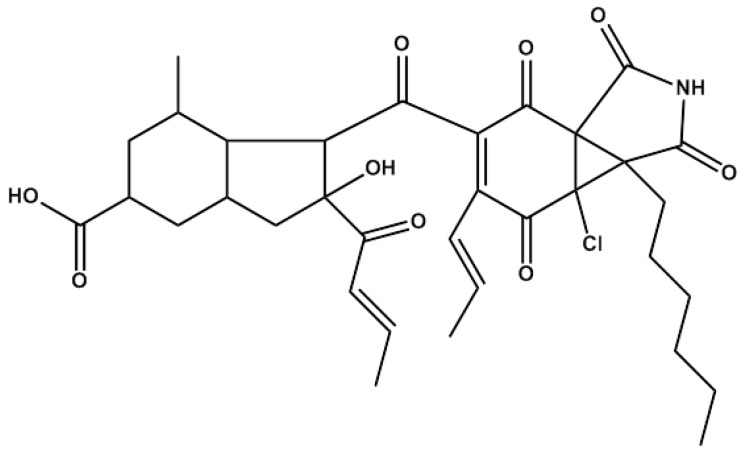
Chemical structure of AD0157.

Marine species are being demonstrated to be an unexplored and prolific source of molecular diversity, yielding an increasing number of products for biotechnological applications, including the production of bioactive compounds for pharmaceutical use [6,7]. Our group is actively involved in the search for new modulators of angiogenesis from marine origin [8,9,10,11]. In the course of a screening program, the pyrrolidinedione, AD0157 (Figure 1), isolated and purified from the fermentation broth of a marine fungi, was selected for its ability to inhibit endothelial cell differentiation *in vitro* [12]. In the present study, we provide evidence that AD0157 is a potent inhibitor of angiogenesis *in vitro*, interfering with several key steps of the angiogenic process. AD0157 inhibits the growth of both endothelial and tumor cells. The growth inhibitory activity of this compound may be related in endothelial cells to an induction of apoptosis. AD0157 reduces the migratory and proteolytic activities of endothelial cells and their ability to form a network of tubular-like structures on Matrigel at micromolar concentrations. The data presented here show that this compound represses the phosphorylation of endothelial serum-stimulated Akt-phosphorylation, suggesting that AD0157 interferes with the molecular mechanisms of cell proliferation, migration and survival. The *in vitro* antiangiogenic effect of AD0157 was confirmed by means of one *ex vivo* and two *in vivo* models. Our results indicate the potential of AD0157 for the treatment of cancer and other angiogenesis-related malignancies and reinforce the concept that marine compounds are a valuable source of new inhibitors of angiogenesis.

## 2. Results and Discussion

### 2.1. AD0157 Inhibits the Growth of Endothelial and Tumor Cells

**Figure 2:**
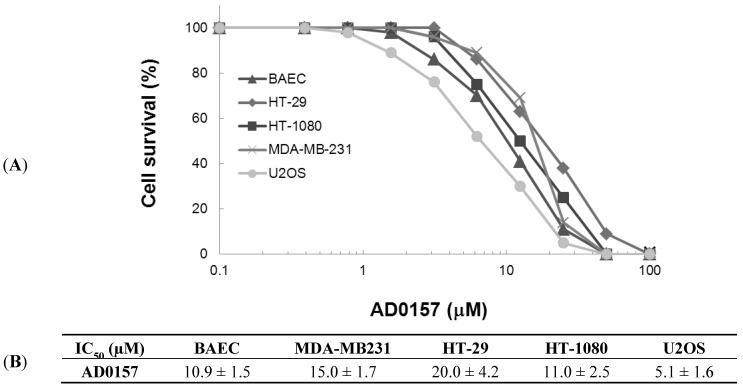
(**A**) Dose-dependent effect of AD0157 on the *in vitro* growth of bovine aortic endothelial cells (BAEC) (▲), MDA-MB-231 (x), HT-29 (♦), HT-1080(■) and U2OS (●). Cell survival is represented as a percentage of control-cell growth in cultures containing no drug. Each point represents the mean of quadruplicates; SD values were typically lower than 10% of the mean values and are omitted for clarity; (**B**) Half-maximal inhibitory concentration (IC_50_) values calculated from dose-response curves as the concentration of compound yielding 50% of control cell survival. They are expressed as means ± SD of three independent experiments.

Angiogenesis involves the local proliferation of endothelial cells. We first investigated the ability of AD0157 to inhibit the growth of serum-activated endothelial and tumor cells. As shown in Figure 2, AD0157 inhibited the growth of cultured bovine aortic endothelial cells (BAECs) with a half-maximal inhibitory concentration (IC_50_) value of 10.9 µM for subconfluent BAECs stimulated to grow with 10% FBS. The data obtained with the HT-1080 fibrosarcoma cell line, HT-29 colon adenocarcinoma cell line, MDA-MB-231 breast carcinoma cell line and U2OS osteosarcoma cell line are in the same range of concentrations as that of BAEC, suggesting that AD0157 is not a specific inhibitor of endothelial cell growth. 

### 2.2. AD0157 Inhibits Capillary Tube Formation by Endothelial Cells

**Figure 3:**
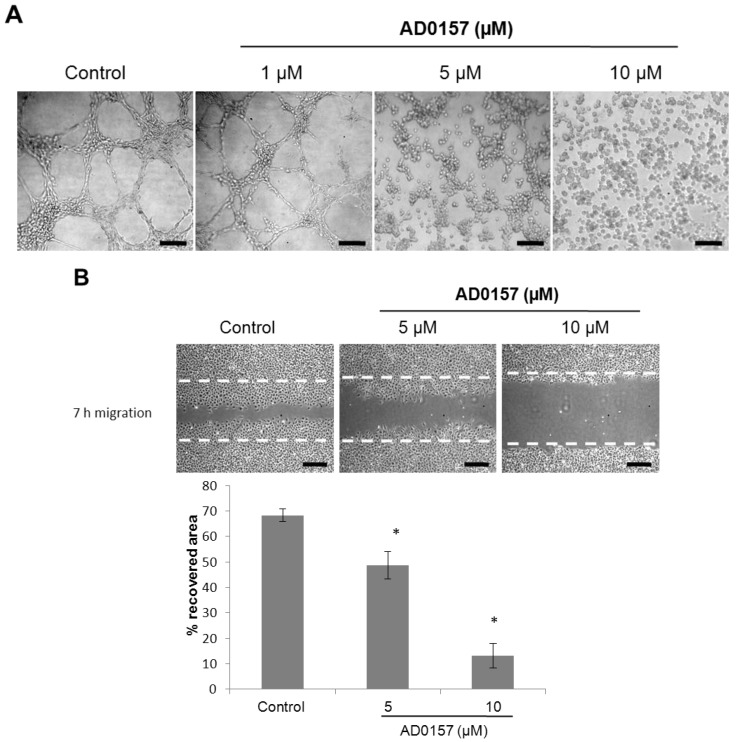
AD0157 inhibits endothelial cell tube formation and migration. (**A**) BAEC seeded on Matrigel formed tubes. AD0157 inhibited endothelial cell tubulogenesis *in vitro* in a dose-dependent manner at non-toxic doses. Cells were photographed 7 h after seeding under an inverted microscope (bar = 100 µm); (**B**) Top panel: confluent BAEC monolayers were wounded, and fresh culture medium was added either in the absence or presence of the indicated concentrations of the compound. Photographs were taken at 7 h of incubation. Broken lines indicate the wound edges (bar = 100 µm); Bottom panel: The regrowth of BAEC into the cell-free area was measured after 7 h, and percentages of the recovered area are expressed as the mean ± SD, * *p* < 0.05 *versus* the control (*n* = 3).

The final event during angiogenesis is the organization of endothelial cells in a three-dimensional network of tubes. *In vitro*, endothelial cells plated on Matrigel align themselves, forming cords, already evident a few hours after plating (Figure 3A, left panel). Figure 3A shows that AD0157, at a concentration of 5 µM or higher, was able to completely inhibit the morphogenesis of endothelial cells on Matrigel, with a partial inhibition of the tube-like structure formation observed at 1 µM. To check the viability of endothelial cells after treatment with the compounds in this assay, BAE cells were incubated in a 96-well plate in the same conditions employed for the tube formation assay. After 7 h, cell viability in comparison to control (untreated cells) was determined by the addition of MTT (3-(4,5-dimethylthiazol-2-yl)-2,5-diphenyltetrazolium bromide), essentially as described for the cell growth assay. The treatment with AD0157, at the concentrations used to inhibit the differentiation of BAE cells, did not affect the viability of those cells after 7 h (results not shown).

### 2.3. AD0157 Inhibits the Migratory Capacity of Endothelial Cells

The acquisition by endothelial cells of the capability to migrate through extracellular matrix is essential for the formation of new blood vessels. To investigate the effect of AD0157 on endothelial cell migration, the so-called “wound healing” assay was used. Figure 3B shows the effects of five and 10 µM AD0157 on endothelial cell migration after 7 h of treatment. Quantitative determination of the invaded area shows a significant dose-dependent inhibition of the migratory capability of BAECs by treatment with subtoxic concentrations of AD0157. 

### 2.4. AD0157 Inhibits the Extracellular Matrix Degrading Potential of Endothelial Cells

**Figure 4:**
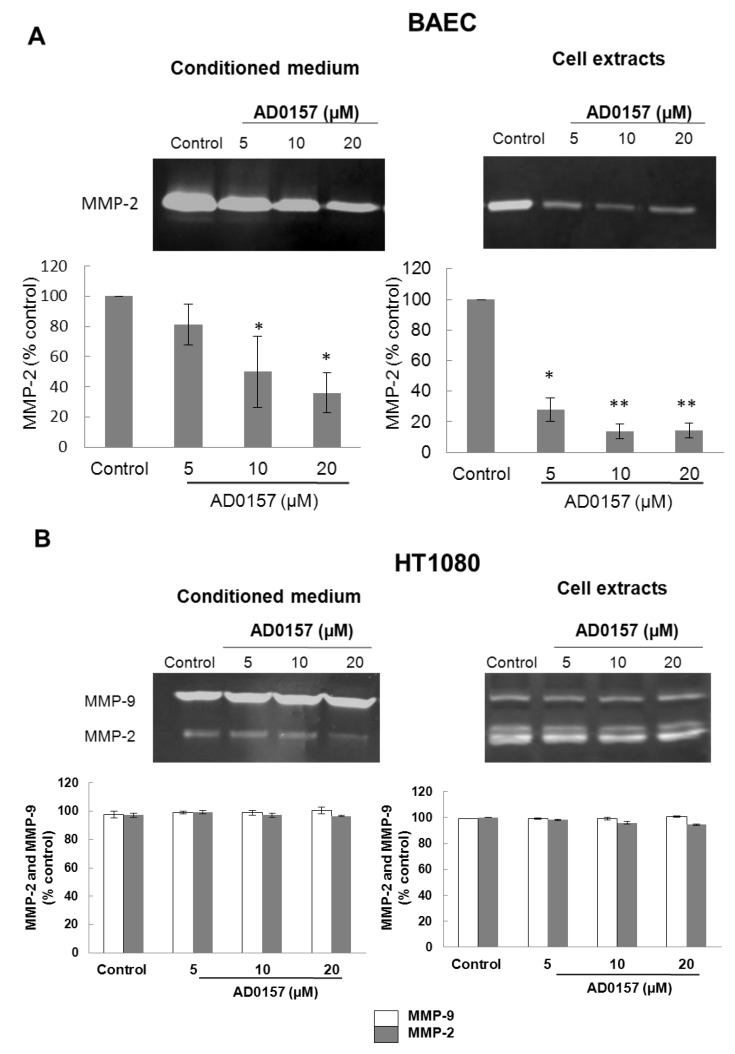
AD0157 inhibits endothelial MMP-2 production. Conditioned media and cellular extracts from BAE cells (**A**) or HT1080 cells (**B**) were treated during 24 h with the indicated concentrations of AD0157, were normalized for equal cell density and used for gelatin zymography. The graphics show the quantification of the normalized relative inhibitory effect on BAEC MMP-2 activity or HT1080 MMP-2 and MMP-9 activities. Data are given as the percentage of the untreated control, and they are the means ± SD of three experimental values. * *p* < 0.05 *versus* the control and ** *p* < 0.005 *versus* the control.

Angiogenesis involves the acquisition by endothelial cells of the capability to degrade the basement membrane and, in general, to remodel the extracellular matrix. MMP-2 (gelatinase A), a matrix metalloproteinase expressed by endothelial cells, plays a relevant role in angiogenesis. Gelatin zymography of conditioned media and cell extracts of BAE cells untreated and treated with different concentrations of AD0157 for 24 h showed that this compound exerted a dose-dependent inhibition of the MMP-2 production by endothelial cells (Figure 4A). Nevertheless, our results show that no effect on the MMP-2 and MMP-9 levels is observed when the HT-1080 tumor cells were treated with AD0157 (Figure 4B), suggesting some endothelial-specificity for the inhibition of the cell proteolytic capabilities by this compound.

### 2.5. AD0157 Inhibits Angiogenesis *in Vivo*

**Figure 5:**
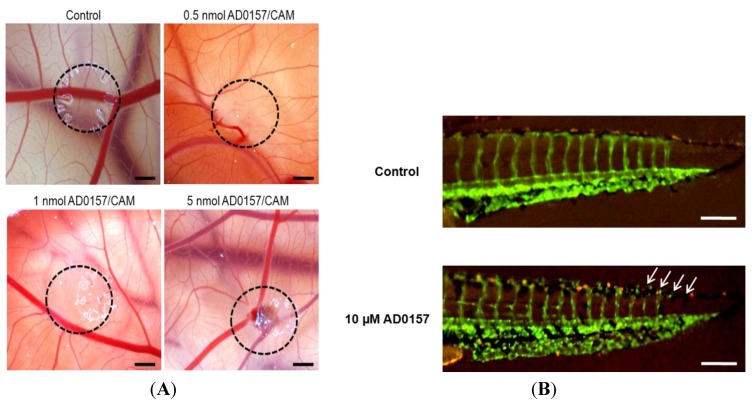
AD0157 inhibits angiogenesis *in vivo.* (**A**) Chorioallantoic membrane assay (CAM). The methylcellulose disc containing the substance vehicle alone and the methylcellulose disc containing 0.5, one and 5 nmol of AD0157. Circles show the locations of the methylcellulose discs (bar = 1 mm); (**B**) Inhibition of the zebrafish neovascularization by AD0157. Transgenic *TG(fli1:EGFP)y1* zebrafish embryos that show green fluorescent protein (GFP) expression in endothelial cells were incubated without or with 10 µM AD0157 (bars represent 50 µm).

The chicken chorioallantoic membrane (CAM) assay, a widely used and accessible system to study angiogenesis, was used to determine the *in vivo* antiangiogenic activity of AD0157 [13]. As shown in Table 1, treatment with AD0157 caused a dose-dependent antiangiogenic effect, which is maintained as low as 0.5 nmol (0.32 µg) per CAM, where 83% of the eggs scored positive, and reaching a maximum activity at 1 nmol (0.64 µg) per CAM, with 100% positive. As shown in Figure 5A, in untreated chorioallantoic membranes (controls), blood vessels form a dense and spatially-oriented branching network composed of vascular structures of progressively smaller diameter as they branch. The inhibition of angiogenesis by AD0157 in this assay was observed as an inhibition of the ingrowth of new vessels in the area covered by the methylcellulose discs. It could also be observed that the peripheral vessels (relative to the position of the disc) grew centrifugally, avoiding the treated area, with an overall decrease in the vascular density. When CAMs were treated with AD0157 at concentrations above 5 nmol (3.2 µg) per CAM, some intratissue hemorrhages in the treated area could be occasionally observed (Figure 5A, bottom right panel). 

**Table 1:** Inhibition of *in vivo* angiogenesis by AD0157.

**CAM assay**	**AD0157 (nmol/CAM)**	**Positive/Total**	**% inhibition**
	0.1	1/5	20
	0.5	5/6	83
	1	6/6	100
	5	6/6	100
**Zebrafish embryo assay**	**AD0157 (µM)**	**Positive/Total**	**% inhibition**
	5	5/20	25
	10	12/25	48
	25	17/20	85

*In vivo* chorioallantoic membrane (CAM) and live fluorescent zebrafish embryo assays were carried out with different doses of AD0157, as described in the Methods section; data are given as the percentage of eggs with inhibited angiogenesis in their CAMs per total number of treated egg CAMs or as the percentage of embryos with inhibited angiogenesis per total number of treated zebrafish embryos.

A second experimental approach used to evaluate the effects of AD0157 on angiogenesis *in vivo* was the use of the model of transgenic zebrafish. Embryos from a transgenic (*TG(fli1:EGFP)y1*) zebrafish line that carries a 15-kb promoter of the transcription factor friend leukemia virus integration-1 (fli-1), which drives the green fluorescent protein (GFP) expression in the endothelium, were treated with different concentrations of AD0157 [14]. Along the development of the zebrafish, intersegmental vessels sprout and grow upward from the aorta, and then, the tips join by anastomosis to form a dorsal vein. Our results show that AD0157, when added to water, inhibited the formation of the last zebrafish intersegmental blood vessels in a dose-response manner, with 85% of zebrafish embryos incubated with 25 µM AD0157 being scored as positive in this assay (Table 1). Inhibition of embryo angiogenesis was observed by a decrease in the width of some vessels and interruptions in the last intersegmental vessels (Figure 5B). The embryos remained viable during the 24-h period of the study, and their overall morphology was similar to control embryos, indicating that development was unaffected and suggesting a low toxicity of this compound.

### 2.6. AD0157 Inhibits Microvessel Outgrowth in Mouse Aortic Rings

**Figure 6:**
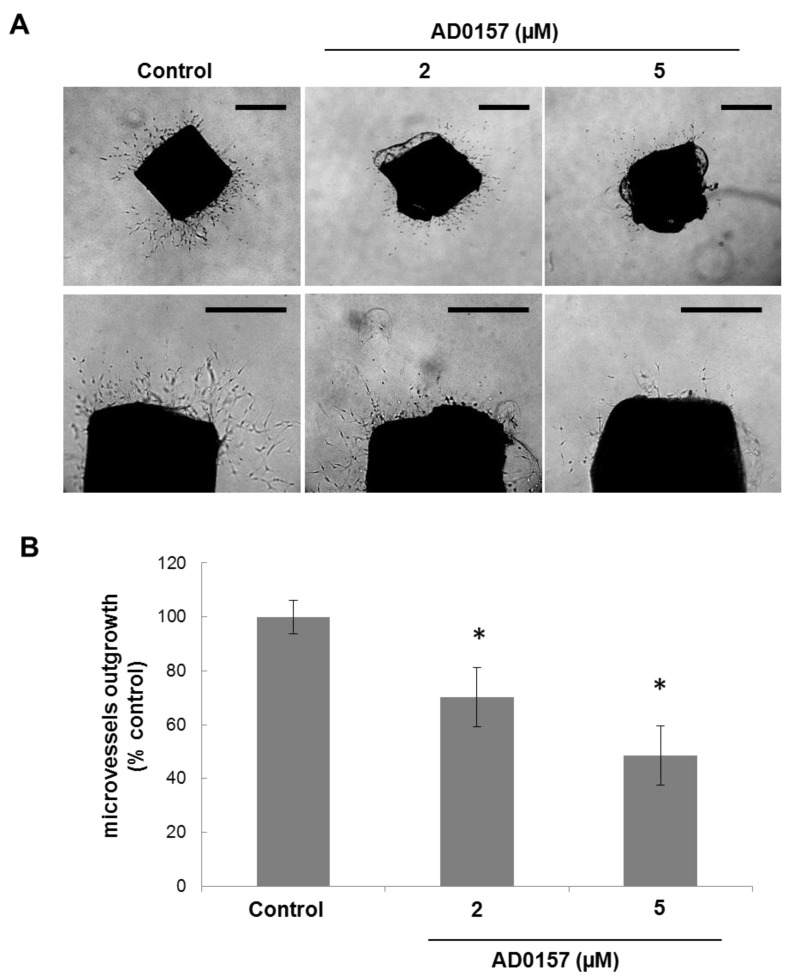
AD0157 inhibits microvessel outgrowth in the mouse aortic ring assay. (**A**) Representative samples of the aortic ring without or with treatment. In the top panel, the whole ring is photographed, and in the bottom panel, only one side is detailed (bar = 500 µm). (**B**) The results were calculated as the area occupied by new microvessels expressed as a percentage by the outgrowth observed in the controls, which we considered as having 100% vessel formation. The results are the mean ± SD of four aortic rings from different mice, * *p* < 0.005 *versus* the control.

An additional line of evidence showing the potential of AD0157 to inhibit overall angiogenesis is provided by the *ex vivo* model of the mouse aortic ring assay [15]. In this assay, the capability of new vessel formation from explants was evaluated after 7 days of incubation of the aortic rings with different concentrations of the compound. As seen in Figure 6A, in the absence of AD0157 (controls), aortic rings were able to generate a dense microvessel outgrowth in a collagen matrix. Our results indicate that AD0157 at concentrations above 2 µM inhibited microvessel formation in a dose-response fashion. 

### 2.7. AD0157 Induces Apoptosis in Endothelial Cells

**Figure 7:**
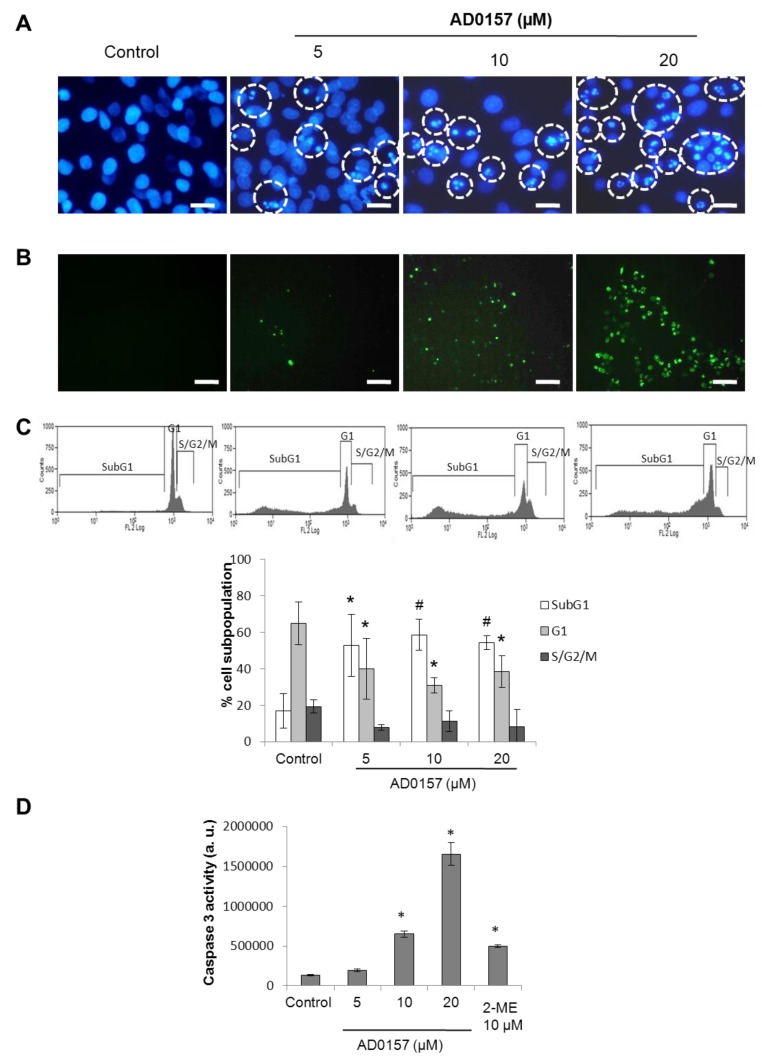
AD0157 induces apoptosis in BAECs. (**A**) AD0157 induces chromatin condensation in endothelial cells (Hoechst staining, bar = 50 µm). Cells with condensed chromatin are highlighted; (**B**) AD0157 induces DNA fragmentation in endothelial terminal deoxynucleotidyl transferase dUTP-biotin nick end-labeling (TUNEL assay, bar = 50 µm); (**C**) The effect of AD0157 on the endothelial cell cycle distribution. After incubation with AD0157, cells were stained with propidium iodide, and the percentage of subG1, G1, and S/G2/M subpopulations were determined using a MoFlo Dako Cytomation cytometer. A representative result and the calculated values for cell subpopulations, expressed as the means ± SD of three independent experiments, are shown, * *p* < 0.05, # *p* < 0.005 *versus* the untreated control; (**D**) The effect of AD0157 on the endothelial cells caspase-3-like activity. Ten micromolar 2-methoxyestradiol (2-ME) was used as a positive control of caspase induction. The results are the mean ± SD of three independent experiments, * *p* < 0.005 *versus* control. In all experiments, BAECs were incubated for 14 h with or without the indicated concentrations of AD0157 in complete medium, as detailed in the Methods section.

As the first approach to determine whether the growth inhibitory activity of AD0157 could be, at least in part, due to the induction of apoptosis, the nuclear morphology of endothelial cells was investigated after 14 h of treatment with different concentrations of this compound. Figure 7A shows that AD0157, at a concentration of 5 µM or higher, induced chromatin condensation in endothelial cells. 

The apoptogenic activity of AD0157 was confirmed by the terminal deoxynucleotidyl transferase mediated dUTP-biotin nick end-labeling (TUNEL) assay, showing that this compound induces DNA fragmentation in BAE cells (Figure 7B). In addition, cell cycle analysis was performed in AD0157-treated BAEC after propidium iodide staining. Flow cytometric analysis showed that AD0157 significantly increased the apoptotic subG1 population at five, 10 and 20 µM (Figure 7C). 

The activation of caspases plays a central role in the induction of apoptosis. To evaluate if endothelial cells caspases could be activated after cell treatment with AD0157, a caspase-3/7 substrate, N-Acetyl-Asp-Glu-Val-Asp-7-amino-4-methylcoumarin or Ac-DEVD-AMC, which is cleaved to a luminescent product by caspase-3, was used. The results shown in Figure 7D indicate that the “effector caspase-3” was significantly activated in a dose-dependent pattern in BAE cells after treatment with AD0157. In this assay, 10 µM 2-methoxyestradiol was used as the positive control of caspase activation. 

### 2.8. AD0157 Interference on the ERK1/2 and Akt Pathways

**Figure 8:**
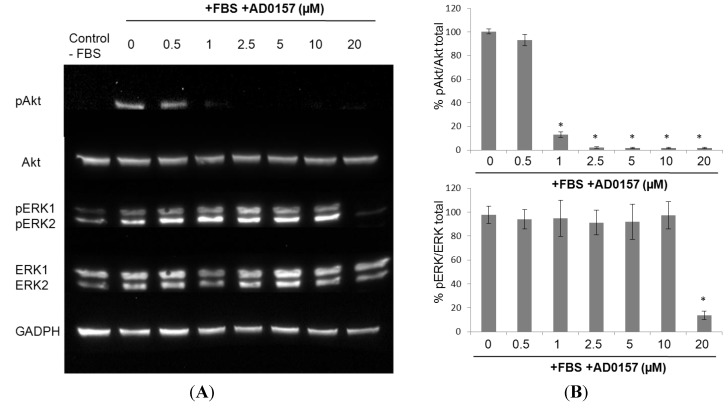
The effect of AD0157 on the ERK1/2 and Akt pathways (**A**) A representative Western blot showing the effects of AD0157 on the content of phosphorylated Akt, phosphorylated ERK-1/2, total Akt and total ERK-1/2 in protein extracts from BAECs; (**B**) Western blots were quantified by densitometry, and the pAkt/total Akt ratio and P-ERK-1/2/ERK-1/2 are expressed as the percentage of the pAkt/total Akt ratio or the P-ERK/total ERK ratio in serum-stimulated BAECs (in the presence of serum, in the absence of AD0157) the means ± SD of four independent experiments), * *p* < 0.001 *versus* the control (in the presence of serum, in the absence of AD0157).

MAPK/ERK1/2 and PI3K/Akt are two of the most relevant signaling pathways controlling angiogenesis [16]. Therefore, we examined the effect of AD0157 on the serum-induced phosphorylation of ERK1/2 and Akt in BAECs. As shown in Figure 8A, treatment of the BAEC with serum resulted in a significant enhancement of both Akt and ERK-1/2 phosphorylation. Akt phosphorylation was significantly inhibited in BAECs by the presence of 1 µM AD0157 (Figure 8B). A similar effect was observed for ERK-1/2 phosphorylation when endothelial cells were serum-activated in the presence of 10 µM AD0157 (Figure 8A). 

### 2.9. Global Discussion

For angiogenesis to take place, activated endothelial cells must proliferate, migrate and invade through the basement membrane and the extracellular matrix and, finally, differentiate to yield a new capillary vessel. Any of these steps can be considered a target for the pharmaceutical inhibition of angiogenesis [5]. 

The vast majority of the natural compounds that have been previously described as inhibitors of angiogenesis have been isolated from plants and terrestrial microorganisms, mainly due to their higher availability and because their therapeutic effects had been previously known in folk traditional medicines [17]. However, increasing attention is being paid to the development of marine-derived antiangiogenic agents, probably fuelled by the increase in the number of marine-derived anticancer drugs, which are being successfully used for cancer therapy [18,19]. Nowadays, it is widely acknowledged that marine organisms produce interesting and singular pharmacological lead compounds, derived from the large diversity of marine habitats and environmental conditions. Marine organisms produce metabolites that allow them to adapt and survive in extreme environments, unique molecules of the highest interest for drug discovery [20]. In this regard, some angiogenesis inhibitors from marine origin have been described by us and others [8,9,10,11,21].

In this paper, we show that AD0157, a pyrrolidinedione isolated from the fermentation broth of a marine fungus, is a new inhibitor of angiogenesis *in vitro*, *in vivo* and *ex vivo*. This compound was first detected in a blind screening of compounds from marine origin that were able to inhibit, at nontoxic doses, the endothelial cell tube formation *in vitro*. AD0157 is able to abrogate the formation of “tubule-like” structures on Matrigel at concentrations that are lower than those required to inhibit endothelial cell growth. Our results also show that similar concentrations of AD0157 produced a significant inhibition of the migratory capability of BAECs, which could contribute to its antiangiogenic activity. These concentrations are in the same range as those required for other previously described inhibitors of angiogenesis, including a number of marine and plant-derived antiangiogenic compounds [8,11,22,23,24,25,26]. Moreover, the mentioned biological activities of AD0157 are exerted at one or two orders of magnitude lower than those required for some other natural compounds that have been previously described as inhibitors of angiogenesis [27,28,29,30]. 

Some of the best characterized anti-angiogenic compounds were initially detected and selected for their capability to interfere with endothelial cell growth, although the desirable endothelial cell specificity of this effect is not a common feature [31]. Our data indicate a non-specific cell growth inhibitory effect in long-term (three days) treatments with micromolar concentrations of AD0157 for endothelial and tumor cells, suggesting that this compound could also behave as a potential anti-tumor drug. The fact that the growth inhibitory activity is exerted at higher concentrations of AD0157 than those required to inhibit the endothelial tube formation suggests that the antiangiogenic activity of this compound is dependent on the prevention of capillary-like tube formation or endothelial cell migration rather than proliferation. 

A positive proteolytic balance is required for capillary sprouting and lumen formation during angiogenesis. Matrix metalloproteinases (MMPs) are essential in the angiogenesis process [32]. MMP-2, also called gelatinase A, is constitutively secreted by endothelial cells contributing to the triggering of tumor angiogenesis *in vitro* and *in vivo* [33,34]. Our data show that incubation with AD0157 inhibits MMP-2 secretion in BAEC-conditioned media and cell lysates, which could contribute to the anti-angiogenic effect of this compound. The inhibition of gelatinases production by AD0157 seems to be endothelial-specific, since no effect on the levels of MMP-2 and MMP-9 (gelatinase B) produced by HT1080 tumor cells was observed after treatment with this compound. Nevertheless, further investigation is needed to confirm and characterize this suggested specificity.

Inhibition by AD0157 of *in vitro* angiogenesis agrees well with the observed effect on the *in vivo* angiogenesis, substantiated by using two widely employed and independent experimental models: the chick chorioallantoic membrane and the live fluorescent zebrafish embryo neovascularization assays. Our data show that AD0157 is a potent inhibitor of angiogenesis *in vivo*, these activities being exhibited in a concentration-dependent fashion at doses that are below those required for other previously described inhibitors of angiogenesis, including the marine natural compounds, aeroplysinin-1, 8-epipuupehedione and toluquinol, among others [8,9,11]. AD0157 *in vivo* antiangiogenic activity was confirmed by means of an *ex vivo* aortic ring assay illustrating the endothelial cell proliferation, migration and the capillary-like tube formation from aortic explants in a collagen matrix [35]. 

Growing evidence indicates that the induction of apoptosis in endothelial cells is a mechanism that could contribute to the biological activity of some antiangiogenic compounds [8,9,10,11]. The results obtained with BAEC indicate that after being exposed to AD0157, endothelial cells exhibit some typical hallmarks of apoptosis, including apoptotic cell morphology and DNA fragmentation. Cell cycle studies clearly show a significant increase in the percentage of cells with sub-diploid DNA content in AD0157-treated BAEC, indicating that the growth inhibitory effect produced by this compound on activated endothelial cells could be due to an induction of apoptosis. Caspases are critical components of the apoptotic machinery [36]. Measurement of the activity of the effector caspase 3 in endothelial cells shows a dose-dependent activation of the caspase proteolytic cascade after treatment with AD0157, suggesting that this compound induces apoptosis in BAEC through a caspase-dependent pathway. 

The angiogenic transformation of the endothelium involves the activation of diverse intracellular signaling pathways. The knowledge about them may provide new targets of antiangiogenic therapies [16]. The PI3K/Akt pathway plays a central role in regulating cell survival, proliferation, migration, regulating normal vascularization and pathological angiogenesis, which makes this pathway an exciting target for molecular therapeutics [37,38]. Our data reveal that AD0157 caused the repression of Akt phosphorylation in serum-stimulated endothelial cells, which indicates that components of this pathway, particularly essential for the survival of the angiogenic endothelium, could be major targets in the molecular mechanism of this compound. The MAPK/ERK1-2 pathway, the main controller of the transduction of proliferation signals, is the molecular target of some successfully developed inhibitors of angiogenesis [39]. The kinase domain phosphorylation of ERK isoforms p44 (ERK-1) and p42 (ERK-2) is stimulated by a wide variety of growth factors and mitogens, including FGF and VEGF [16]. Although our results show that incubation with AD0157 abrogates the serum-stimulated ERK1-2 phosphorylation, the fact that this activity is only observed at concentrations that are ten times higher than those required to inhibit Akt phosphorylation is in agreement with the cell cycle studies showing that this compound is preferentially acting on endothelial cell survival, rather than on endothelial proliferation. Nevertheless, deactivation of the ERK pathway in activated endothelial cells by high concentrations of AD0157 could explain the observed downregulation of MMP-2, mediated by the mentioned signaling pathway [40]. 

## 3. Experimental Section

### 3.1. Materials

Cell culture media, penicillin, streptomycin and amphotericin B were purchased from Biowhittaker (Walkersville, MD, USA). Fetal bovine serum (FBS) was a product of Harlan-Seralab (Belton, UK). Matrigel was purchased from Becton–Dickinson (Bedford, MA, USA). Plastics for cell culture were supplied by NUNC (Roskilde, Denmark) and VWR (West Chester, PA, USA). Hanks’ Balanced Salt Solution (HBSS) and MCDB131 medium were obtained from Gibco (Grand Island, New York, NY, USA). Collagen was provided by SERVA Electrophoresis (Heidelberg, Germany). Fertilized chick eggs were obtained from Granja Santa Isabel (Córdoba, Spain). The antibodies used in this work were purchased from Cell Signaling Technology (Danvers, MA, USA). Supplements and other chemicals not listed in this section were obtained from Sigma Chemicals Co. (St. Louis, MO, USA). AD0157, kindly supplied by Biomar S.A. (León, Spain), was purified as a colorless powder from the mycelium of a fungal strain, *Paraconiothyrium* sp. HL-78-gCHSP3-B005, isolated from a marine Chordatasample collected in Guatemala, by extraction with EtOAc/MeOH [12]. The organic extract was purified by VFC (vacuum flash chromatography), flash-column chromatography on silica gel and by reverse phase HPLC. Data regarding the fermentation of this fungus, extraction, isolation and structural determination of AD0157 are available as Supplementary Information.

### 3.2. Cell Culture

Bovine aortic endothelial cells (BAEC) were isolated from bovine aortic arches, as previously described [41], and maintained in Dulbecco’s modified Eagle’s medium (DMEM) containing glucose (1 g/L), glutamine (2 mM), penicillin (50 IU/mL), streptomycin (0.05 mg/mL) and amphotericin (1.25 mg/L) supplemented with 10% FBS (DMEM/10% FBS). Human fibrosarcoma HT-1080 cells were maintained in DMEM containing glucose (4.5 g/L), glutamine (2 mM), penicillin (50 IU/mL), streptomycin (0.05 mg/mL) and amphotericin (1.25 mg/L) supplemented with 10% FBS. Human colon adenocarcinoma HT-29 cells and human osteosarcoma U2OS cells were maintained in McCoy’s 5A medium containing glutamine (2 mM), penicillin (50 IU/mL), streptomycin (50 µg/mL) and amphotericin (1.25 µg/mL) supplemented with 10% FBS. Human breast cancer carcinoma MDA-MB-231 was maintained in RPMI1640 medium (medium developed at Roswell Park Memorial Institute) containing glutamine (2 mM), penicillin (50 IU/mL), streptomycin (50 µg/mL) and amphotericin (1.25 µg/mL) supplemented with 10% FBS. All cell lines were maintained at 37 °C and humidified 5% CO_2_ atmosphere.

### 3.3. Cell Growth Assay

The 3-(4,5-dimethylthiazol-2-yl)-2,5-diphenyltetrazolium bromide or MTT dye reduction assay in 96-well microplates was used. This assay is dependent on the reduction of MTT by mitochondrial dehydrogenases of a viable cell to a blue formazan product, which can be measured spectrophotometrically. Three times ten to the power of three BAEC, 2 × 10^3^ HT-1080, HT-29, MDA-MB-231 and U2OS cells in a total volume of 100 µL of their respective growth media were incubated with serial dilutions of AD0157. After 3 days of incubation (37 °C and 5% CO_2_ in a humid atmosphere), 10 µL of MTT (5 mg/mL in phosphate-buffered saline (PBS)) were added to each well, and the plate was incubated for a further 4 h at 37 °C. The resulting formazan was dissolved in 150 µL of 0.04 N HCl/2-propanol and read at 550 nm. All determinations were carried out in quadruplicate, and three independent experiments were carried out. IC_50_ values were calculated as those concentrations of AD0157 yielding 50% cell survival, taking the values obtained for the control to be 100%.

### 3.4. Endothelial Cell Differentiation Assay: Tube Formation on Matrigel

Wells of 96-well microplate were coated with 50 µL of Matrigel (10.5 mg/mL) at 4 °C and allowed to polymerize at 37 °C for a minimum of 30 min. Some 5 × 10^4^ BAE cells were added in 200 µL of complete medium. Finally, different amounts of AD0157 were added and incubated at 37 °C in a humidified chamber with 5% CO_2_. After incubation for 7 h, cultures were observed and photographed with a Nikon DIAPHOT-TMD inverted microscope (Tokyo, Japan). Each concentration was tested in duplicate, and two different observers evaluated the results of chord formation inhibition. Those assays where no tubular structure could be observed were considered as positive in this assay.

### 3.5. Endothelial Cell Migration Assay

The migratory activity of BAEC was assessed using a wounded migration assay. Confluent monolayers in 6-well plates were wounded with pipet tips with 2 perpendicular diameters, giving rise to 2 acellular 1 mm-wide lanes per well. After washing, cells were supplied with 1.5 mL complete medium in the absence (controls) or presence of different concentration of AD0157. Wounded areas were photographed. After an additional 4, 7 and 24 h of incubation, plates were observed under the microscope, and photos were taken of the same areas as those recorded at time zero. The amount of migration at 7 h was determined by image analysis in both controls and treated wells and normalized with respect to their respective values at time zero, using the NIH Image 1.6 software. The regrowth of BAEC into the cell-free area was calculated as the percentage of the initial wounded area (time 0) that had been recovered by endothelial cells after 7 h.

### 3.6. Gelatinolytic Activity

To prepare conditioned media and cell lysates, BAEC and HT1080 cells were grown at 75% confluency in 6-well plates. After 2 washes with PBS, each well received the indicated concentration of AD0157 in 1.5 mL of DMEM/0.1% bovine serum albumin (BSA) containing 200 kallikrein inhibitor units (KIU) of aprotinin/mL. After 24 h of incubation, the conditioned media were collected, and the cells were washed twice with PBS and harvested by scrapping into 0.5 mL of 0.2% Triton X-100 in 0.1 M Tris/HCl containing 200 KIU of aprotinin. Duplicates were used to determine the cell number by using a Coulter counter. The media and cell lysates were centrifuged at 1000× *g* at 4 °C for 20 min, and the supernatants were collected and used for assayed gelatinolytic activity.

The gelatinolytic activities of MMP-2 and MMP-9 delivered to the conditioned media or present in cell lysates were detected in gelatinograms. Aliquots of conditioned media and cell lysates normalized for equal cell numbers were subjected to non-reducing SDS-PAGE with gelatin (1 mg/mL) added to the 10% resolving gel. After electrophoresis, gels were washed twice with 50 mM Tris/HCl, pH 7.4, supplemented with 2% Triton X-100, and twice with 50 mM Tris/HCl, pH 7.4. Each wash was for 10 min and with continuous shaking. After the washes, the gels were incubated overnight at 37 °C immersed in a substrate buffer (50 mM Tris/HCl, pH 7.4, supplemented with 1% Triton X-100, 5 mM CaCl_2_ and 0.02% Na_3_N). Then, the gels were stained with Commassie Blue R-250, and the bands of gelatinase activity could be detected as non-stained bands in a dark, stained background. Quantitative analysis of gelatinograms was performed with the NIH Image 1.6 program.

### 3.7. Chorioallantoic Membrane (CAM) Assay

Fertilized chick eggs were incubated horizontally at 38 °C in a humidified incubator, windowed by day 3 of incubation and processed by day 8. The compound AD0157 were added to a 1.2% solution of methylcellulose in water, and 10 µL drops of this solution were allowed to dry on a Teflon-coated surface in a laminar flow hood and implanted on the CAM. After 48 h reincubation, the CAM was examined under a stereomicroscope by two different observers. The assay was scored as positive when both of them reported a significant reduction of vessels in the treated area [8].

### 3.8. Zebrafish Embryo Assay

Transgenic fli-EGFP fish (*TG(fli1:EGFP)y1*) had a vasculature labeled with GFP and were purchased from the Zebrafish International Resource Center (ZIRC, Eugene, OR, USA). Zebrafish embryos were generated via natural pairwise mating and maintained in embryo water at 28.5 °C. Embryos were manually dechorionated with forceps at 24 h postfertilization (hpf), arrayed in a 96-well plate (one embryo per well) and incubated with different concentrations of AD0157 in 100 µL of water at 28.5 °C for 24 h. DMSO was used as both the carrier of drugs and control. After incubation, fish embryos were anesthetized with tricaine (0.02%), placed on slides and examined under an epifluorescence Nikon microscope equipped with a DS-L1 Nikon (Chiyoda-Ku, Tokyo, Japan) digital Leica DM IL inverted microscope (Leica Microsystems, Wetzlar, Germany) at low and high magnification [27].

### 3.9. Mouse Aortic Ring Assay

Thoracic aortas were removed from 2 to 4 month-old C57BL/6 mice and immediately transferred to a culture dish containing Dulbecco’s modified Eagle’s medium (DMEM). The periaortic fibroadipose tissue was carefully removed with fine microdissecting forceps and iridectomy scissors paying special attention not to damage the aortic wall. One-millimeter aortic rings (approximately 15 per aorta) were sectioned and embedded in a rat tail interstitial collagen gel (1.5 mg/mL) prepared by mixing 7.5 volumes of 2 mg/mL collagen, 1 volume of 10× Hank’s Balanced Salt Solution (HBBS), 1.5 volume of 186 mM NaHCO_3_ and 0.1 volume of 1 M NaOH to adjust the pH to 7.4. The collagen gels containing the aortic rings were polymerized in cylindrical agarose wells and kept in quadruplicate at 37 °C in 60 mm diameter Petri dishes (bacteriological polystyrene, Falcon, Becton Dickinson, Lincoln Park, NJ, USA). Each Petri dish contained 6 mL of MCDB131 medium supplemented with 1% l-glutamine, 25 mM NaHCO_3_, 100 U/mL penicillin, 100 µg/mL streptomycin and 2.5% mouse serum, in the presence or absence of AD0157 or DMSO. The cultures were kept at 37 °C in a humidified environment for a week and examined every second day with a phase contrast microscope (Zeiss, Axiovert 25) at appropriate magnification. 

The quantification of aortic rings was done with NIH Image 1.6 software. The results were calculated as the area occupied by new microvessels expressed as a percentage by the outgrowth observed in the controls, which we considered to have 100% vessel formation. Four aortic rings from different mice were quantified in each experimental condition.

### 3.10. Apoptosis and Cell Cycle Assays

For Hoechst staining experiments, cells were plated on 8-well chamber slides and grown to sub-confluence. After treatments for 14 h with the indicated concentrations of AD0157 in complete medium, cells were washed with PBS and fixed with formalin solution. Chamber slides were stained with 1 mg/mL Hoechst in PBS for 2 min, washed twice with PBS and mounted (Dako Cytomation Fluorescent Mounting Medium, Denmark). Samples were observed under a fluorescence microscope (Leica, TCS-NT, Heidelberg, Germany).

For DNA fragmentation studies, cells were grown to sub-confluence in complete medium on 8-well Falcon humidified chamber slides and incubated for 14 h with or without the indicated concentrations of AD0157. After incubation, cells were washed with PBS, fixed with formalin solution, washed with PBS again and permeabilized with 0.1% Triton X-100 in PBS. The TUNEL (terminal deoxynucleotidyl transferase mediated dUTP-biotin nick end-labeling) assay was performed with the use of the *In Situ* Cell Death Detection Kit (Roche Diagnostics, Barcelona, Spain), according to the manufacturer’s instructions. Finally, cells were mounted using Dako Cytomation Fluorescent Mounting Medium and observed under a fluorescence microscope (Leica, TCS-NT).

For cell cycle analysis, BAECs were grown in complete medium in a 6-well plate to 75% confluency. After treatment with different concentrations of AD0157 during 14 h, attached and detached cells were harvested and centrifuged at 1000× *g*. Pellets were washed whit PBS and resuspended in 250 µL of ice-cold PBS. For fixation, 70% ice-cold ethanol was added while continuous gentle vortexing, and samples were maintained on ice for 1 h. Finally, cells were centrifuged and washed twice with PBS, resuspended in 500 µL propidium iodide staining solution (40 µg/mL propidium iodide and 0.1 mg/mL RNase-A in PBS) and incubated for 1 h with shaking and protection from light. Percentages of the subG1, G1 and S/G2/M populations were determined using a MoFlo Dako Cytomation cytometer and the software, Summit 4.3.

For the determination of caspase 3/7 activity, BAECs were plated in 96-well plates (13,000 cells/well) and treated with or without different concentrations of AD0157 for 14 h. Then, Caspase-Glo^®^ 3/7 reagent (Promega Biotech Ibérica, Madrid, Spain) was added to the wells, according to the manufacturer’s instructions, and the luminescence was recorded at 30 minutes with a GLOMAX 96 microplate luminometer (Promega Biotech Ibérica, Madrid, Spain). The assay provides a proluminescent caspase-3/7 DEVD-aminoluciferin substrate and a proprietary thermostable luciferase in a reagent optimized for caspase-3/7 activity, luciferase activity and cell lysis.

### 3.11. Western Blot Analysis

Subconfluent BAEC cultures were incubated in DMEM medium supplemented with 1% fetal bovine serum (FBS) for 24 h. After that, the medium was replaced, and the cells were incubated with different doses of AD0157 or vehicle (DMEM) for 30 min and, then, challenged for 20 additional minutes with FBS or the vehicle (DMEM medium). The protein lysates were obtained by scrapping the cells in a lysis buffer (50 mM Tris, pH 7.4, 150 mM NaCl, 1% Triton X-100, 0.25% sodium deoxycholate, 1 mM EDTA, 1 mM sodium orthovanadate and 5 mg/mL of a protease inhibitors mixture). Afterwards, the extracts were centrifuged at 13,000 rpm for 15 min at 4 °C, evaluated for protein concentration by the Bradford test and stored at −80 °C until the moment of analysis. These samples were denatured for 5 min at 95 °C and subjected to SDS-PAGE, loading some 20 µL of sample per well. After electrophoresis, samples were electrotransferred to nitrocellulose membranes, blocked with 5% dried skimmed milk in 50 mM Tris pH 8.4, 0.9% NaCl, 0.05% Tween 20 (Tris buffered saline-Tween 20, TBST) and incubated overnight in the presence of an anti-human phosphorylated Akt or an anti-human phosphorylated ERK-1/2 at a dilution of 1:1000 in TBST or an anti-human total Akt or an anti-human total ERK-1/2 at a dilution of 1:1000 in TBS-T with 5% BSA. After three washing steps with TBST, the membranes were incubated with horseradish peroxidase-conjugated anti-rabbit secondary antibody at a dilution of 1:5000 in blocking buffer for 2 h at room temperature. After three washing steps with TBST, the immunoreactive bands were detected using a chemiluminescence system (SuperSignal West Pico Chemiluminescent Substrate, Pierce, Rockford, IL, USA) with an imaging system (Chemidoc XRS System, Bio-Rad, Hercules, CA, USA) and were quantified by using ImageLab version 3.0 software. The membranes were incubated with an anti-GAPDH primary antibody at a dilution of 1:1000 to ensure equal loading. Akt or ERK-1/2 activation was calculated as the p-Akt/total Akt and p-ERK-1/2**/**ERK-1/2 ratio and expressed as the mean ± SD of 3 independent experiments.

### 3.12. Statistical Analysis

All data are expressed as the means ± standard deviation (SD). A one-tailed Student’s *t*-test was used for evaluations of the pairs of means, to establish which groups differed from the control group. *p* < 0.05 was considered to be statistically significant.

### 3.13. Animal Testing Regulations

Experimentation animals were kept at the animal house facilities of the University of Málaga. Experiments on animals were subjected to the European Directive 86/609/EEC on the protection of animals used for experimental and other scientific purposes, under the approval of the Bioethics Committee of the University of Málaga.

## 4. Conclusions

In conclusion, in this paper, we show evidence that the marine pyrrolidinedione, AD0157, is a potent inhibitor of angiogenesis *in vitro*, *in vivo* and *ex vivo*. AD0157 abrogates certain functions of endothelial cells, namely, differentiation, migration, growth and proteases production. The induction of endothelial cell apoptosis by AD0157 could be related to an inhibition of the Akt signaling pathway in activated endothelial cells. Although additional studies will be needed to elucidate the molecular mechanisms underlying the antiangiogenic activity of AD0157 and the pharmaceutical toxicological profile of this compound, the data presented here suggest a potential therapeutic application of AD0157 for the treatment of angiogenesis-related malignances. These data reinforce the great value of marine products as candidates for further pharmaceutical studies for feeding the anti-angiogenesis drug pipeline. Further efforts to determine the pharmacological potential of AD0157 are warranted.

## Supplementary Files

Supplementary File 1 Supplementary Information (PDF, 114 KB)
