# Supplementary material for: The Marine Fungal Metabolite, AD0157, Inhibits Angiogenesis by Targeting the Akt Signaling Pathway
Source: Mar Drugs. 2014 Jan 16;12(1):279–99. doi: 10.3390/md12010279 (PMC3917274; doi:10.3390/md12010279)
Supplement: Supplementary File 1 — Supplementary Information (PDF, 114 KB) [file marinedrugs-12-00279-s001.pdf]

Supplementary Information

S1. Experimental Section

S1.1. Specimen Collection and Identification of the Marine-Derived Fungus Paraconiothyrium sp. *HL-78-gCHSP3-B005*

Compound AD0157 (Figure S1) was purified from the culture broth of the marine fungus, *Paraconiothynium* sp. HL-78-gCHSP3-B005, which was isolated from an unidentified marine Chordata sample collected in Guatemala. A culture of the strain has been deposited in the Colección Española de Cultivos Tipo at the Universidad de Valencia, Spain under the accession number CECT 20841. Taxonomical determination was confirmed after a sequencing analysis of ITS1-5.8S-TS2 ribosomal DNA region. The sequence showed a similarity percentage of 95% with the sequence of *Paraconiothyrium variabile*. Conidia characteristics and a low similarity value did not confirm the species level, but allowed us to identify the isolate as *Paraconiothyrium* sp. Colonies reached 7 cm in diameter in 10 days at 25 °C on potato dextrose agar in a culture chamber that maintains a humidity of 42%. The optimal temperature for growth on solid media is 24–28 °C and the pH range for growth is between five and seven. Growth was best with glucose and starch. A pure culture of *Paraconiothyrium* sp. HL-78-gCHSP3-B005 was kept frozen at −70 °C in 20% glycerol.

S1.2. Fermentation, Extraction and Isolation of AD0157 from Paraconiothyrium *sp. HL-78-gCHSP3-B005*

A well grown agar culture was used to inoculate 40 mL of seed medium containing 2% oatmeal, 2% malt extract, 0.01% KH_2PO_4, 0.005% $MgSO_4$, and tap water in 250 mL shake flasks and cultured at 24 °C on a rotary shaker at 200 rpm. The flasks were incubated 48 hours in the dark, and used as a first stage inoculum. 250 ml of the same medium in 2 L Erlenmeyer flasks were inoculated with 10% of the first stage inoculum. The fermentation was carried out for seven days at 24 °C on a rotary shaker at 200 rpm in the dark. Production of this compound was monitored by HPLC.

The fermentation broth (4.5 L) of the fungus HL-78-gCHSP3-B005 was filtered through celite and the mycelial cake was extracted twice with 2 liters of a mixture of EtOAc/MeOH (3:1). The resultant suspension was filtered and partitioned between EtOAc and water. The organic layer was taken to dryness and the crude extract (9.31 g) was fractionated by VFC (vacuum flash chromatography) on silica gel, eluted with a stepwise gradient of hexane/EtOAc/MeOH. Fractions containing compound AD0157 (eluted with EtOAc/MeOH 9:1, 225 mg) were applied to a silica gel column and flash-chromatographed by elution with a $CHCl_3$/MeOH gradient. Fractions containing compound AD0157 (eluted with $CHCl_3$/MeOH 93:7, 83 mg) were finally purified by semipreparative reversed-phase HPLC, affording 23 mg of a colorless powder of pure compound AD0157.

HPLC analysis was performed at room temperature using an analytical Symmetry C18 column (5 µm, 3.9 × 150 mm) and as a mobile phase gradient from 50% MeOH/H2O (1% formic acid) to 100% MeOH for 20 min and 100% MeOH for 10 min more, a flow rate of 0.7 mL/min. and plotted at 220 nm, in these conditions the retention time for Compound AD0157 was 17.90 min.

The molecular formula of compound AD0157 was determined to be $C_{34}H_{40}NO_9Cl$ from the HPLC-APCI-MS (pseudomolecular ion at *m/z* of 642 $[M + H]^+$ and an isotopic peak at *m/z* of 644 with

a ratio of 3:1) and from HRESI–MS (found *m/z* 642.2493 $[M + H]^+$, calcd. *m/z* 642.2464 for $C_{34}H_{41}NO_9Cl$ and found *m/z* 640.2320 $[M - H]^-$, calcd. *m/z* 640.2319 for $C_{34}H_{39}NO_9Cl$), which was supported by the number of carbons in the ^{13}C NMR spectrum (Table S1). The IR spectrum showed broad strong bands from 1630 to 1700 cm^{-1} suggesting the presence of several carbonyl functions. The complete assignments of ^1H and ^{13}C NMR spectra of compound AD0157 were finally established by 2D NMR experiments COSY, HSQC and HMBC and all the spectroscopic data are shown in Table S1.

Figure S1. Structure of AD0157.

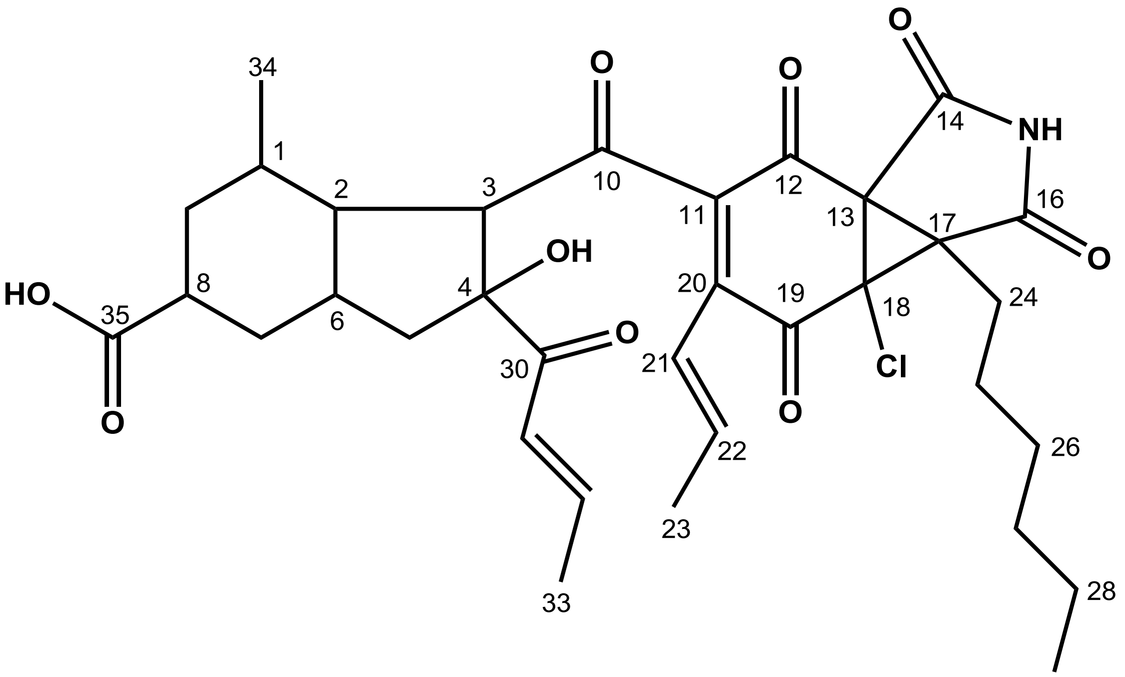

Table S1. ^1H and ^{13}C NMR data of **AD0157** in CD_3OD.

Position	^{13}C (δ)	^1H (δ)	HMBC correlations
1-CH	36.8	1.48 (1H, m)	
2-CH	51.4	1.83 (1H, d, 10.5)	C1, C2, C6, C7, C10
3-CH	61.2	4.10 (1H, d, 10.5)	C1, C2, C4, C10, C30
4-C	86.2		
5-CH_2	45.4	1.46 (1H, m)	C-4, C6, C30
		2.05 (1H, m)	C2, C3, C6, C30
6-CH	41.8	1.42 (1H, m)	C5
7-CH_2	33.4	1.20 (1H, m)	C2, C6 C35
		2.06 (1H, m)	C2, C6
8-CH	43.3	2.40 (1H, m)	C34
9-CH_2	38.3	1.22 (1H, m)	
		1.93 (1H, m)	C1, C7, C8, C34, C35
10-CO	195.7		
11-C	145.4		

Table S1. *Cont.*

Position	^{13}C (δ)	^{1}H (δ)	HMBC correlations
12-CO	186.4		
13-C	49.7[a]		
14-CO	168.6		
15-NH	10.40[b]	(1H, brs)	C13, C14, C16, C17
16-CO	171.9		
17-C	48.0[a]		
18-C	53.9[a]		
19-CO	191.1		
20-C	148.2		
21-CH	119.0	6.52 (1H, dq, 16.0, 1.7)	C11, C18, C19
22-CH	147.2	7.48 (1H, dq, 16.0, 7.0)	C19
23-CH_3	19.6	2.02 (3H, dd, 7.0, 1.7)	C20, C21
24-CH_2	21.7	206 (1H, m)	C13, C16, C18, C25, C26
		2.27 (1H, ddd, 13.8, 11.7, 4.23)	C13, C16, C18, C25, C26
25-CH_2	27.3	1.05 (1H, m)	C26, C27
		1.71(1H, m)	C26, C27
26-CH_2	29.1	1.28 (2H, m)	C27
27-CH_2	31.2	1.26 (2H, m)	C25, C28, C29
28-CH_2	22.3	1.28 (2H, m)	C27, C29
29-CH_3	13.2	0.92 (3H, t, 7.1)	C27, C28
30-CO	201.6		
31-CH	125.0	6.68 (1H, dq, 15.4, 1.6)	C30
32-CH	145.7	6.92 (1H, dq, 15.4, 6.8)	C30
33-CH_3	17.6	1.90 (3H, dd, 6.8, 1.6)	C30, C31, C32
34-CH_3	20.8	0.81 (3H, d, 6.6)	C1,C2, C9
35-CO	178.3		
4′-OH		3.84 [b] (1H, s)	C-3, C-4, C-5, C30

[a] Interchangeable signals; [b] acetone–D6 spectra.
